# A highly sensitive nanobody-based immunoassay detecting SARS-CoV-2 nucleocapsid protein using all-recombinant reagents

**DOI:** 10.3389/fimmu.2023.1220477

**Published:** 2023-07-11

**Authors:** Paula Segovia-de los Santos, Carolina Padula-Roca, Ximena Simon, Cesar Echaides, Gabriel Lassabe, Gualberto Gonzalez-Sapienza

**Affiliations:** ^1^ Cátedra de Inmunología, Departamento de Biociencias (DEPBIO), Facultad de Química, Instituto de Higiene, Montevideo, Uruguay; ^2^ ATGen SRL, Montevideo, Uruguay; ^3^ Parque Lecocq, Intendencia Municipal de Montevideo (IMM), Montevideo, Uruguay

**Keywords:** COVID-19, testing, nanobody, in-house, NanoLuc, nucleocapsid protein, SARS-CoV-2, luminescent ELISA

## Abstract

Antigen tests have been crucial for managing the COVID-19 pandemic by identifying individuals infected with SARS-CoV-2. This remains true even after immunity has been widely attained through natural infection and vaccination, since it only provides moderate protection against transmission and is highly permeable to the emergence of new virus variants. For this reason, the widespread availability of diagnostic methods is essential for health systems to manage outbreaks effectively. In this work, we generated nanobodies to the virus nucleocapsid protein (NP) and after an affinity-guided selection identified a nanobody pair that allowed the detection of NP at sub-ng/mL levels in a colorimetric two-site ELISA, demonstrating high diagnostic value with clinical samples. We further modified the assay by using a nanobody-NanoLuc luciferase chimeric tracer, resulting in increased sensitivity (detection limit = 61 pg/mL) and remarkable improvement in diagnostic performance. The luminescent assay was finally evaluated using 115 nasopharyngeal swab samples. Receiver Operating Characteristic (ROC) curve analysis revealed a sensitivity of 78.7% (95% confidence interval: 64.3%-89.3%) and specificity of 100.0% (95% confidence interval: 94.7%-100.0%). The test allows the parallel analysis of a large number of untreated samples, and fulfills our goal of producing a recombinant reagent-based test that can be reproduced at low cost by other laboratories with recombinant expression capabilities, aiding to build diagnostic capacity.

## 1 Introduction

The knowledge in late 2019 that a highly infectious novel coronavirus, SARS-CoV-2, had begun circulating in the human population marked the beginning of one of the largest global public health crises that humanity has faced in recent times. Diagnostic methods for detecting acute infection were a critical component in managing and controlling the pandemic. Indeed, timely identification of infected individuals to manage their isolation and avoid transmission was essential in the early days of the pandemic when the population was immunologically naïve to the infection (1), and continues to be so because the immunity generated through natural infection and vaccination, although of enormous value to reduce the impact of severe disease and mortality, has shown a moderate effect in preventing transmission and is particularly permeable to the emergence of new variants of the virus (2, 3).

Viral culture has been proposed as the most reliable way to establish whether an individual is infectious, but the technique is complex, highly specialized, and requires very high levels of biosafety. In addition, different studies have revealed the difficulty in demonstrating the presence of the virus by this technique, which translates into a much lower sensitivity than that achieved by molecular methods of nucleic acid amplification such as the quantitative reverse transcription polymerase chain reaction (RT-qPCR) (4, 5). Since the start of the pandemic, the analysis of nasopharyngeal or oro-pharyngeal samples by RT-qPCR became the reference standard diagnostic method, using particular cycle threshold (Ct) values to classify individuals as potentially infected (6, 7). Intrinsic to the method, the detection of viral nucleic acids by RT-qPCR works with a very low false positive rate and is extremely sensitive (8), but its instrumentation requires expensive equipment, costly reagents, and specialized personnel. In addition, it has been shown that Ct values and their correlation with viral load have a significant degree of variation between laboratories, even when the target genes are the same (9, 10).

Although less sensitive than RT-qPCR, assays that detect the presence of viral antigens (antigen tests) are much cheaper, can be portable, require less equipment, and are easy to use with minimal requirements for sample processing. The antigen of choice in most antigen-detection assays is the nucleocapsid protein (NP), a structural protein whose primary function is to package the viral RNA genome to form the nucleocapsid. Additionally, coronaviral NPs have been shown to play regulatory roles, being involved in viral genome replication (11, 12) and in the perturbation of host cellular processes (13). NP is a 46 kDa protein and consists of an N-terminal domain and a C-terminal domain, linked by an intrinsically disordered serine/arginine-rich region (14). Its high expression level (15, 16) allows for the development of more sensitive assays compared to those that target other viral proteins such as Spike (17). Furthermore, because of its lower mutation rate, antibodies against NP are more likely to react with the NP of emerging virus variants (18, 19). These factors make it the most suitable option for antigen detection tests.

NP detection tests have their detection peak four days after the onset of symptoms and their sensitivity increases with a second test after 1 or 2 days in the early stage of infection (5). Its diagnostic peak largely overlaps with the period of highest viral load and therefore it correlates with the highest period of infectiousness (20). For these reasons, antigen tests began gradually to occupy a very important place in the control of the pandemic, initially as a complementary entry test to RT-qPCR, and later, in many cases, as the frontline method used for the diagnosis of infection and discharge management of patients (21, 22). As a consequence of the global impact of the pandemic, a huge number of commercial antigen tests have been developed, mostly lateral flow immunoassays. Diagnostic parameters vary significantly among tests. A review by the Cochrane group of 20 commercially available tests found that average sensitivities ranged from 34.3% to 91.3% in symptomatic participants, while the specificity was generally high, with 17 of 20 tests meeting the WHO acceptable performance criterion of 97% specificity (23).

During outbreaks, the load of samples to be processed can be overwhelming, which can become a critical bottleneck for patient care and epidemic control. For this reason, the aim of this work was to generate a simple yet sensitive laboratory antigen test that allows the parallel analysis of a high number of untreated samples, based on recombinant reagents that can be produced locally. To achieve this goal, we chose to work with nanobodies (Nbs) as immunodetection elements. Nanobodies are the recombinant form of the variable domain of the heavy chain-only antibodies (HcAbs) found in camelids (family *Camelidae*) and have emerged as highly advantageous diagnostic reagents. They can be produced inexpensively as soluble protein expressed in the *Escherichia coli* periplasm (24), and possess high affinity and outstanding stability (25). Their single-domain nature allows the construction of rich phage display libraries with full preservation of the specificity generated *in vivo*. This is a major advantage over conventional antibody libraries where the random combination of heavy and light chains during library construction makes it difficult to recover the original specificity (25). This comprehensive representation of the immunization-induced immune repertoire allows for the application of different forms of selective pressure throughout panning of the library, thus identifying Nbs with the desired functionality. Taking advantage of this feature, we have previously developed a high-throughput strategy for the selection of nanobody pairs that enable highly sensitive detection of biomarkers by sandwich immunoassays (26). In this work, we generated a Nb phage display library from a llama immunized with the nucleocapsid protein of SARS-CoV-2 and performed a pairwise selection of two nanobodies for the detection of the antigen in nasopharyngeal swabs. To maximize sensitivity, shorten assay time and facilitate the in-house preparation of all reagents, the detection Nb was fused through a flexible linker to NanoLuc, a small (19 kDa) luciferase enzyme derived from the deep-sea shrimp *Oplophorus gracilirostris* (27). NanoLuc has been engineered to be stable, soluble, highly expressed, and to use affordable substrates, displaying a 150-fold higher specific activity than other available luciferases (27). In our application, the Nb-NanoLuc chimera resulted not only in a reduction in test time, but also in a considerable increase in the diagnostic value of the test compared to the colorimetric detection.

## 2 Materials and methods

### 2.1 Materials

D-biotin, isopropyl β-D-1-thiogalactopyranoside (IPTG), LB Broth (Miller), trypsin from bovine pancreas, 3,3’,5,5’-tetramethylbenzidine (TMB), Tween 20, polyethylene glycol 8000 (PEG), and other common chemicals were purchased from Sigma-Aldrich (St. Louis, MO, USA). The anti-hemagglutinin epitope (anti-HA) antibody conjugated to horseradish peroxidase (HRP) was also from Sigma-Aldrich (Cat No. 12013819001). Antibiotics were from AppliChem (Darmstadt, Germany). Bovine Serum Albumin (BSA) was from Golden West BioSolutions (Temecula, CA, USA). TRIzol reagent and streptavidin were from Invitrogen (Carlsbad, CA, USA). Lymphocyte Separation Media (density 1.077 g/mL), Dulbecco’s Modified Eagle Medium (DMEM) and Fetal Bovine Serum (FBS) were from Capricorn Scientific (Ebsdorfergrund, Germany). Molecular biology reagents, *E. coli* One Shot BL21(DE3) cells and antibiotic-antimycotic solution for cell culture were purchased from Thermo Fisher Scientific (Waltham, MA, USA). PEI MAX was from Polysciences (Warrington, PA, USA). *E. coli* ER2738 electrocompetent cells were purchased from Lucigen Corporation (Middleton, WI, USA). Helper phage M13KO7 was purchased from New England Biolabs (Ipswich, MA, USA). Plasmid extraction, PCR clean-up and gel extraction kits were purchased from Qiagen (Germantown, MD, USA). ELISA strips and plates and 96-deep-well culture blocks were from Greiner Bio-One (Monroe, NC, USA). SARS-CoV-2 BA.5 nucleocapsid protein was from Acro Biosystems (Newark, DE, USA). Chromatography columns were from Cytiva (Uppsala, Sweden). Bio-Layer Interferometry Amine Reactive Second-Generation (AR2G) biosensors, N-hydroxysuccinimide (NHS), 1-ethyl-3-(3dimethylaminopropyl) (EDC) and ethanolamine solution were from ForteBio Inc. (Menlo Park, CA, USA). Furimazine was from CSNpharm (Arlington Heights, IL, USA). SnapGene software was used for the design of genes and primers (from Insightful Science, available at snapgene.com). Primers and genes were obtained from General Biosystems Inc. (Morrisville, NC, USA).

### 2.2 Expression and purification of nucleocapsid protein

SARS-CoV-2 full-length nucleocapsid protein (NP) (Genbank, Gene ID: 43740575) and NPΔ121 (an N-terminal deletion mutant lacking the conserved residues 1-121) were cloned into the pET-28a(+) expression plasmid. NP contained a C-terminal Strep-tag, while NPΔ121 contained a C-terminal 6xHis tag and AviTag peptide (a target for site-specific biotinylation by *E. coli*’s biotin ligase). Plasmids were electroporated into either *E. coli* BL21(DE3) or *E. coli* BL21(DE3)-pBir cells respectively (pBir cells carry the pCY216 vector for overexpression of *E. coli*’s biotin ligase BirA). Flasks containing 200 mL LB-40 μg/mL kanamycin (supplemented in the case of *E. coli* BL21(DE3)-pBir cells with 100 μM biotin, 35 μM chloramphenicol, and 0.04% arabinose to induce expression of the biotin ligase) were inoculated with 2 mL of an overnight culture started from a single colony. Expression was induced at OD_600 nm_ = 0.6 with 10 μM IPTG and cultures were grown overnight at 28°C. The following day, cells were harvested by centrifugation and resuspended in 50 mM Tris, 500 mM NaCl, 1 mM PMSF and 10 μg/mL RNAse A, pH 7.5, supplemented with 1 mM EDTA in the case of NP culture. Cells were lysed by sonication. NPΔ121 cell lysates were supplemented with 1 mM biotin and incubated for 2 hours at 37°C with shaking to allow efficient biotinylation. After obtaining cell lysate supernatants by centrifugation, NP was purified using a StrepTrap XT column, while NPΔ121 was purified using a Ni-NTA column, in both cases according to the manufacturer’s instructions and through the ÄKTA purification system (GE Healthcare, Uppsala, Sweden).

### 2.3 Llama immunization and phage display library construction

A 3-year-old llama (*Lama glama*) from Lecocq Municipal Park Zoo (Montevideo) was immunized by subcutaneous injection with 3 doses of 500 µg of full-length SARS-CoV-2 NP in incomplete Freund adjuvant (one dose every 15 days). Ten days after the final booster 200 mL of blood were drawn and peripheral blood mononuclear cells were obtained by centrifugation on Lymphocyte Separation Media (density 1.077 g/mL) gradients. Total RNA from 6 × 10^7^ cells was extracted using TRIzol reagent and reverse-transcribed using RevertAID reverse transcriptase and the HcAb hinge-specific primers INQ-H2 (5’-GGTTGTGGTTTTGGTGTCTTGGGTT-3’) and INQ-H3 (5’-GAGCTGGGGTCTTCGCTGTGGTGCG-3’), which allow the reverse-transcription of the variable domain of HcAbs (mostly VHH domains, occasionally HcAb-associated VH domains) but not of conventional antibodies. cDNA of these variable domain genes was amplified by polymerase chain reaction (PCR) as previously described (28), SfiI-digested, cloned into the pComb3X phagemid vector and electroporated into *E. coli* ER2738 cells. To generate the phage library, transformed cells were cultured and superinfected with helper phage M13KO7. The next day, after harvesting the supernatant by centrifugation, phage particles were obtained by precipitation with 20% polyethylene glycol 8000 as previously described (28).

### 2.4 Panning for the selection of NP-specific nanobodies

Four wells of a high-binding 8-well strip were coated with 100 μL/well of 1 μg/mL full-length NP by overnight incubation at 4°C. After blocking with PBS-1% BSA for 30 minutes at 37°C, wells were incubated with 1 × 10^10^ colony-forming units of the phage library for 1.5 hours at room temperature (RT). After 10 rounds of washing with PBS-0.05% Tween 20 (PBS-T), a 30-minute incubation with PBS-T at RT and a further 10 rounds of washing with PBS-T, bound phages were eluted by adding 50 μL/well of 10 mg/mL trypsin in TBS buffer and incubating for 30 min at 37°C. The phage output was titrated and amplified in *E. coli* ER2738 for a second round of selection. Two rounds of panning were conducted in this way. For the third round of panning, two strategies were carried out in parallel, the first one as described above (non-competitive strategy), and the second one including a competition step prior to elution, where bound phages were incubated with 100 μL/well of 50 μg/mL NP overnight at 4°C. The latter strategy is intended to promote the selection of clones with a slow kinetic dissociation constant (k_off_), since clones with a faster k_off_ would at some point dissociate from the immobilized antigen and be captured by the excess antigen in solution.

### 2.5 High-throughput expression of nanobodies and screening

DNA from the final output of the non-competitive panning strategy was amplified by using 50 μL of phage output to infect 500 μL of an *E. coli* ER2738 culture (OD_600 nm_ = 1.0), which was then diluted in 9.5 mL of SB broth supplemented with 100 μg/mL ampicillin and grown overnight at 37°C. Phagemid DNA was isolated using the QIAprep^®^ Spin Miniprep Kit, SfiI-digested and gel-purified using the QIAquick^®^ Gel Extraction Kit. The purified nanobody genes were cloned into the SfiI-digested pINQ-HAH6 vector (29), which allows the expression of HA- and 6xHis-tagged Nbs. The ligation product was electroporated into *E. coli* BL21(DE3) cells, and 92 individual colonies were cultured in 500 μL of LB medium containing 40 μg/mL kanamycin in a 96-deep-well culture block. Nb expression was induced at OD_600 nm_ = 0.6 with 10 μM IPTG and cultures were incubated overnight at 37°C. The next day, pellets were harvested by centrifugation, resuspended in 200 μL PBS and lysed by four freeze−thaw cycles followed by 30 min of sonication in a sonicator bath. Cell lysates were centrifuged, and supernatants were later used to test Nb reactivity to NP.

Expression of Nbs from the final output of the slow k_off_ selection strategy was performed by inoculating 2 mL cultures in SB broth with 100 μg/mL ampicillin with isolated colonies containing the phagemid vector and inducing Nb expression at OD_600 nm_ = 0.6 with 1 mM IPTG overnight at 37°C. In this case, culture media supernatant was used directly for screening. Screening was carried out by ELISA, incubating cell lysates or cell culture supernatants on wells coated with either full-length NP or streptavidin followed by biotinylated NPΔ121 and blocked with PBS-1% BSA. Bound Nbs were detected using an anti-HA-HRP conjugate.

### 2.6 Expression and purification of selected nanobodies

Three selected Nb clones were SfiI-digested and cloned into two different vectors for bacterial expression: pINQ-HAH6, which allows the expression of HA- and 6xHis-tagged Nbs, and pINQ-BtH6 (28), which allows the expression of 6xHis-tagged Nbs containing also the AviTag peptide. pINQ-HAH6 clones were electroporated into *E. coli* BL21(DE3) cells, and pINQ-BtH6 clones into *E. coli* BL21(DE3)-pBir cells. Cell cultures and site-specific biotinylation were done as described above for NP and NPΔ121. Expression was induced at OD_600 nm_ = 0.6 with 1 μM IPTG and cultures were grown overnight at either 20 or 28°C (conditions were previously optimized for each clone). The following day, cells were harvested by centrifugation, resuspended in PBS or PBS-1 mM biotin and lysed by sonication. Cell lysate supernatants were supplemented with 300 mM NaCl and 20 mM imidazole and Nbs were purified by Ni-NTA columns using the ÄKTA purification system. Finally, Nbs were eluted with 250 mM imidazole, dialyzed against PBS and kept at -20°C until use.

For the expression of the Nb-NanoLuc fusion protein, the ON10 Nb was cloned into the pcDNA3.1(+) vector for transient expression in HEK293T cells. The expression cassette consisted of the Igκ leader sequence for protein secretion, the nanobody gene in tandem with NanoLuc luciferase and the Twin-Strep-tag. Cells were cultured in DMEM supplemented with 10% fetal bovine serum and antibiotic-antimycotic, at 37°C and 5% CO_2_. Cell cultures at approximately 80% confluence were transfected by using PEI MAX in a 5:1 PEI:DNA mass/weight ratio. Culture media supernatant was harvested by centrifugation 4 days later, and the protein was purified using a StrepTrap XT column according to the manufacturer’s instructions.

### 2.7 K_D_ determinations and epitope binning by Bio-Layer Interferometry

Binding of Nbs to NP was studied by Bio-Layer interferometry using the BLItz system (ForteBio, Inc., Menlo Park, CA, USA) and Amine Reactive Second-Generation (AR2G) biosensors. Biosensors activated with N-hydroxysuccinimide (NHS) and 1-ethyl-3-(3-dimethylaminopropyl)carbodiimide (EDC) were incubated with 39 μg/mL NP and then blocked with 1 M ethanolamine, pH 8.5. For K_D_ determinations, a baseline step was carried out in 400 μL of 10X kinetic buffer (PBS with 0.2% Tween 20, 1% BSA and 0.05% sodium azide). Nb association was measured on the drop holder for 120 s at concentrations of 10, 25, 50, 100, 200 and 400 nM in 10X kinetic buffer, followed by dissociation in 400 μL of 10X kinetic buffer for 120 s. All steps were carried out with shaking at 2200 rpm. Data were globally fitted to a 1:1 binding ratio model for calculating the kinetic parameters using Blitz Pro Software, version 1.2 (ForteBio Inc., USA).

For epitope binning, each Nb clone was bound separately at 100 μg/mL in consecutive 120 s-steps, with shaking at 2200 rpm. As each new clone was bound, all previous clones were included in the solution to counteract the potential displacement of bound Nbs.

### 2.8 Nanobody sandwich ELISA for the detection of NP

ELISA wells (clear for colorimetric ELISA and white for luminescent ELISA) were coated with 100 μL of 2 μg/mL streptavidin diluted in PBS for 1 hour at 37°C and blocked with 200 μL of 1% casein and 0.05% Tween 20 in carbonate-bicarbonate buffer pH 9.6 for 30 minutes at 37°C. Biotinylated Nbs were immobilized by incubating 100 μL of 4 μg/mL Nbs overnight at 4°C. The next day, wells were incubated for 1 hour at RT with either 100 μL of serial dilutions of recombinant NP in ATGen’s Viral Transport Medium (VTM), generously provided by ATGen, or 100 μL of nasopharyngeal swab sample in VTM. In colorimetric assays, NP was detected using an HA-tagged Nb followed by an anti-HA-HRP conjugate, each incubated for 1 hour at RT, and finally TMB substrate. Absorbance was read at 450 nm with Fluostar Optima reader (BMG, Ortenberg, Germany). In luminescent assays, wells were incubated with the NbON10-NanoLuc fusion protein for 1 hour at RT, followed by addition of 10 μM furimazine substrate in PBS containing 1% Triton X-100, 0.25 mg/mL BSA and 8.8 mM EDTA, pH 8.0. This buffer, described by Ren et al (30), allows a good balance of luminescent intensity and signal half-life. The substrate was incubated for 1 minute with shaking and signal was read with Fluostar Optima reader, with an integration time of 0.5 s/well. For both colorimetric and luminescent assays, wells were washed six times with PBS-T after each step.

For each assay, titration curves were constructed using serial dilutions of full-length NP to determine analytical sensitivity. Data was fitted by lineal regression using GraphPad Prism 7 and the limit of detection was defined as the mean absorbance value of the blank plus three standard deviations (31).

### 2.9 Clinical samples

Nasopharyngeal swabs were collected in VTM and RT-qPCR-tested by ATGen. The samples analyzed in this study were leftover specimens that were anonymized by encoding, so that the identity of the subjects remained anonymous to all persons associated with the research. Triton X-100 was added to a final concentration of 0.5% for viral inactivation (32), and samples were stored at -20°C until their use. In February 2022 n=19 positive and n=10 negative samples were collected, when Omicron (B.1.1.529) had already become the dominating variant in Uruguay, representing nearly 100% of new cases that month (33). A further n=47 positive and n=68 negative samples were collected during March and April 2022, when Omicron B.1.1529 continued to be the dominant variant.

## 3 Results

### 3.1 The selection of nanobodies was designed to isolate high affinity clones against both NP and NPΔ121

A phage display nanobody library of 1 × 10^7^ transformants was constructed from 6 × 10^7^ peripheral blood mononuclear cells (PBMCs) of a llama that was previously immunized with SARS-CoV-2 full-length nucleocapsid protein (NP) (Genbank, Gene ID: 43740575). Specific nanobodies were selected by performing three rounds of panning on high binding 8-well strips coated with NP. The nanobody gene pool from the final phage output was cloned *en masse* into the pINQ-HAH6 vector for the expression of HA- and 6xHis-tagged nanobodies, and after electroporation into *E. coli* BL21(DE3) individual colonies were picked and grown in a 96-deep-well culture block. A screening was carried out on ELISA plates coated with either full-length NP or NPΔ121, a truncated version of NP that lacks a highly conserved region in the N-terminal domain (NTD) (34, 35). Since nanobodies that react with NTD would be more likely to cross-react with NPs from other human coronaviruses, leading to false positives in diagnosis, we aimed to select nanobody candidates that reacted with NPΔ121, in order to ensure the specificity of our sandwich ELISA by including at least one of them. At this stage, 92 clones were screened and the top ten clones with the highest readouts at high dilution (10^-3^) against either NP or NPΔ121 (indicating a high affinity and/or expression level) were selected (**Supplementary Figure 1**).

Using the phage output from the second round of panning as a starting point, another round of selection was performed in parallel to promote the selection of high affinity clones, by including an overnight competition step where bound phages were exposed to excess antigen in solution. Thus, lower k_off_ clones that eventually dissociated could react with the excess of soluble antigen and be washed off. Ten clones from the resulting phage output (named ON1 to ON10) were screened as described above, four of which were strongly positive (data not shown).

**Figure 1:**
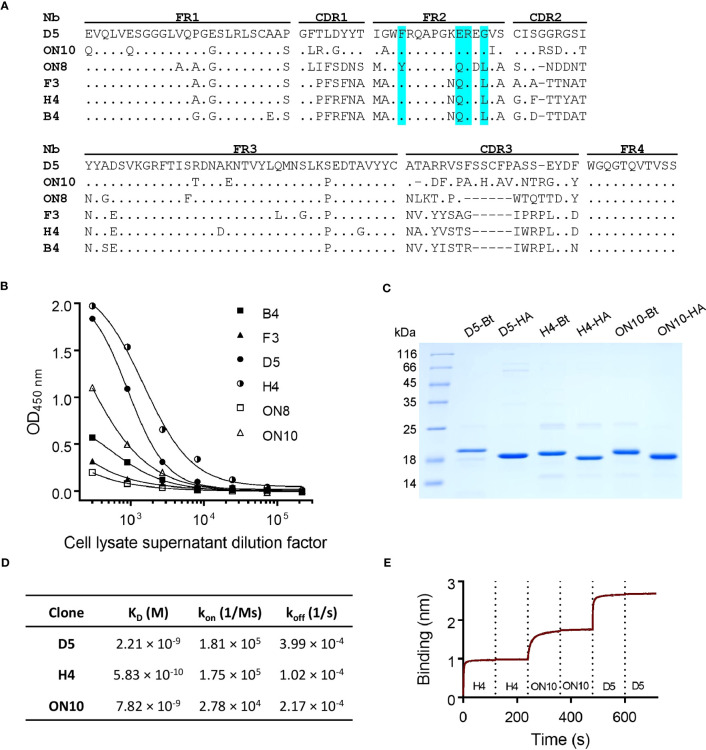
Anti-NP nanobody selection from phage display library, production and characterization. **(A)** Nanobody amino acid sequence alignment of six unique sequences. Dots represent identity and dashes represent gaps. The Framework (FR), Complementarity-Determining Regions (CDR) and hallmark residues of VHH (cyan) are shown. Nucleotide sequences are available at GenBank accession numbers OQ982376, OQ982375, OQ982379, OQ982378, OQ982374 and OQ982377, corresponding to D5, ON10, ON8, F3, H4 and B4 respectively. **(B)** Titration of cell lysate supernatants of six different clones on ELISA plates coated with NP. Higher reactivity represents greater affinity and/or relative expression levels. **(C)** SDS-PAGE of purified nanobodies. -Bt and -HA represent site-specifically biotinylated or HA-tagged nanobodies respectively. **(D)** Affinity and kinetic constants determined by Bio-Layer Interferometry (BLI) using immobilized NP. **(E)** Epitope binning BLI sensogram using immobilized NP. Each nanobody was included twice consecutively to ensure binding sites had been saturated.

Through sequence analysis of the selected clones from both outputs, six unique sequences were found (**Figure 1A**), all of which possessed in framework 2 (FR2) the hallmark residues of VHH domains (as opposed to VH domains), i.e. F/Y42, E/Q49, R50 and F/G/L52 (36). The sequences appear to correspond to four different germ lines (F3, H4 and B4 seem to have diversified by somatic hypermutation). D5 presents two additional cysteine residues, most probably forming an extra disulfide bridge. Interestingly, F3 presents a putative N-glycosylation site in its CDR2, which could be a source of variability if it were to be expressed in mammalian cells. Protein expression of these clones was induced in 2 mL *E. coli* cultures, and cell lysate supernatants were titrated on ELISA plates coated with NP (**Figure 1B**). The clones with the highest relative affinity/expression level were selected, namely D5, H4 and ON10. In order to further characterize these clones and test them as potential pairs for the antigen-capture ELISA, the expression conditions were optimized for each one and they were produced on a larger scale and purified in two versions, either HA-tagged or site-specifically biotinylated through the use of AviTag (**Figure 1C**). Yields of purified protein ranged from 4.0 to 18.5 mg per liter of *E. coli* culture and were sequence-dependent.

### 3.2 The three selected nanobodies define non-overlapping epitopes and have nM affinity

Equilibrium dissociation constants (K_D_) were determined by Bio-Layer Interferometry using the BLItz system (ForteBio). All three clones were shown to bind NP with high affinity, especially H4, with a determined K_D_ of 5.83 × 10^-10^ M compared to 2.21 × 10^-9^ M for D5 and 7.82 × 10^-9^ M for ON10 (**Figure 1D**; **Supplementary Figure 2**). Although the panning strategy for ON10 was aimed at selecting clones with a slow k_off_ rate, its K_D_ was hindered by a slow k_on_ rate. Nevertheless, the use of both strategies broadened the repertoire of NP-specific nanobody sequences, which is important for the empirical optimization of two-site assays.

Epitope binning was performed by sequential exposure of the immobilized antigen on the biosensors to saturation concentrations of each nanobody. We found that the epitopes of the three clones did not overlap, as nanobodies could bind sequentially to their antigen regardless of the order in which they were added (**Figure 1E**; **Supplementary Figure 3**), indicating that these three clones could potentially constitute capture/detection pairs in a sandwich ELISA.

### 3.3 After pairwise selection, an NP colorimetric ELISA with a detection limit below ng/mL was obtained

**Figure 2:**
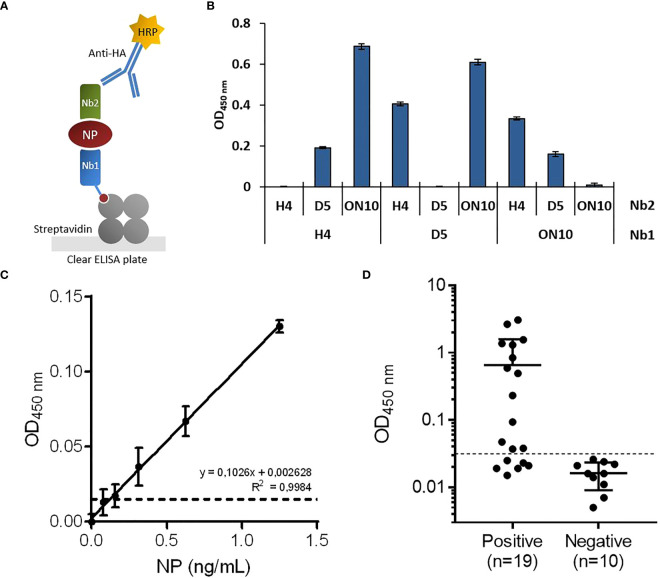
Development of antigen-capture ELISA. **(A)** Schematic representation. Biotinylated nanobodies (Nb) are orientedly immobilized on plates coated with streptavidin and the captured NP is detected with HA-tagged nanobodies followed by an HRP-conjugated anti-HA monoclonal antibody. **(B)** Determination of the best capture/detection nanobody pair. Nine possible combinations using the three selected nanobodies were tested by sandwich ELISA with 5 ng/mL of NP. **(C)** Antigen detection with the selected capture/detection nanobody pair (H4/ON10). Serial dilutions of full-length NP were analyzed by triplicate; data are plotted as mean ± SD. The dashed line represents the limit of detection, calculated as the mean absorbance value of the blank plus 3×SD. Linear regression was performed using GraphPad Prism 7. **(D)** NP detection in nasopharyngeal swab samples classified as positive (n=19) or negative (n=10) by prior RT-qPCR analysis.

As our group and others have previously reported (37–39), coating ELISA plates directly with nanobodies often results in inefficient antigen capture, presumably because their small size means that their structure and therefore also their antigen binding capability is compromised when they are adsorbed to the plate. To overcome this problem, biotinylated nanobodies were immobilized in streptavidin-coated plates, ensuring in addition a more favorable spatial orientation of capture nanobodies. After the antigen-capture step, NP was detected using an HA-tagged nanobody followed by an anti-HA-HRP conjugate antibody (**Figure 2A**).

To determine the best capture/detection nanobody pair, all possible combinations were tested (**Figure 2B**). None of the pairs generated background signal, as measured in the absence of NP. The H4/ON10 pair produced the highest readout and was therefore selected to establish the NP antigen-capture ELISA. The nucleotide sequences of the cassettes used to produce the biotinylated Nb H4 and the HA-tagged Nb ON10 are shown in **Supplementary Figures 4**, **5**, respectively (the corresponding plasmids are available at Addgene IDs 198690 and 198689). The H4 clone exhibited the highest affinity against NP, and it is specific for the less conserved C-terminal region (as it reacted with NPΔ121). Therefore, we expected it to contribute to generating not only a sensitive but also a highly specific assay.

**Figure 3:**
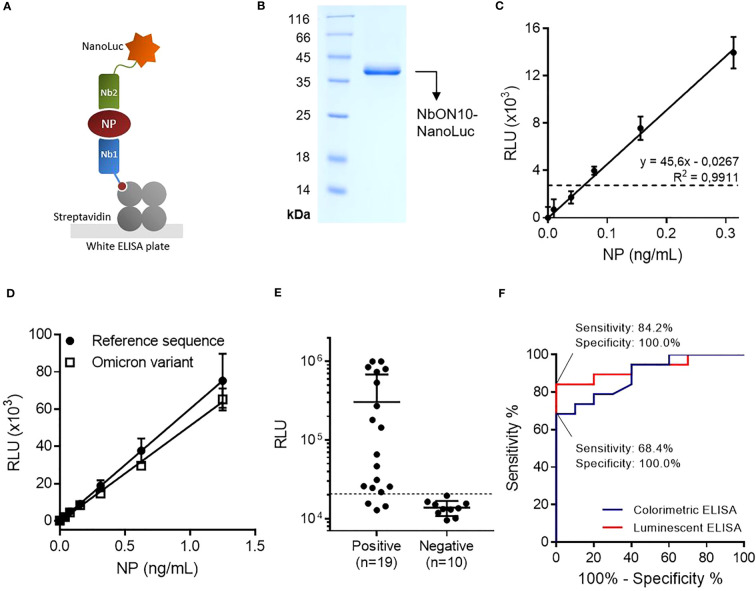
Highly sensitive antigen-capture assay developed using a nanobody-NanoLuc fusion for detection. **(A)** Schematic representation. Biotinylated nanobodies (Nb) are orientedly immobilized on plates coated with streptavidin and the captured NP is detected with a nanobody fused to NanoLuc luciferase. **(B)** SDS-PAGE of purified NbON10-NanoLuc. **(C)** Antigen detection with luminescent antigen-capture ELISA. Serial dilutions of full-length NP were analyzed by quadruplicate; data are plotted as mean ± SD. The dashed line represents the limit of detection, calculated as the mean absorbance value of the blank plus 3×SD. Linear regression was performed using GraphPad Prism 7. **(D)** Detection of NP of the ancestral and Omicron BA.5 SARS-CoV-2 variants. The BA.5 variant protein carries the mutations P13L, Δ31-33, E136D, R203K, G204R and S413R, a group that includes all the mutations found in other common Omicron variants. **(E)** NP detection in nasopharyngeal swab samples (n=19 positive and n=10 negative samples classified by prior RT-qPCR analysis). The same sample set was previously analyzed by colorimetric ELISA (**Figure 2D**). **(F)** Receiver Operating Characteristic (ROC) curves for colorimetric and luminescent antigen-capture ELISA.

Titration curves were constructed using serially diluted full-length NP, and the limit of detection was determined to be 121 pg/mL (**Figure 2C**). Next, we evaluated the assay’s performance in relation to NP detection in clinical specimens. A group of previously RT-qPCR tested nasopharyngeal swab samples were analyzed, consisting of n=19 positive samples and n=10 negative samples. After a cutoff value was established through a Receiver Operating Characteristic (ROC) curve by setting the specificity as 100.0% (95% confidence interval (CI): 69.2%-100.0%; **Figure 3F**), the assay presented a sensitivity of 68.4% (95% CI: 43.5%-87.4%), classifying as positive 13/19 of the RT-qPCR-positive samples (**Figure 2D**).

### 3.4 The simplicity and diagnostic power of the test were greatly improved by fusing the detection nanobody to NanoLuc

With the aim of improving the assay’s sensitivity, we decided to develop a luminescent ELISA by producing the detection nanobody fused to NanoLuc (**Figure 3A**), a small (19 kDa) and highly stable luciferase that produces an intense signal with excellent dynamic stability. First, we attempted to produce the NbON10-NanoLuc fusion in *E. coli* BL21(DE3). In previous work, our group had produced nanobody-NanoLuc chimeras in *E. coli* using unrelated nanobodies (unpublished work), however in this case expression was unsuccessful. A wide range of expression conditions were explored, including different culture media, a range of temperatures (20-37°C) and inducer concentrations (IPTG 1-1000 μM), and codon optimization through the use of two different online servers (Genewiz, from Azenta Life Sciences, available at genewiz.com, and IDT Codon Optimization Tool, from Integrated DNA Technologies, available at idtdna.com). Although the protein was highly expressed, it was present exclusively in the form of inclusion bodies (data not shown). Refolding from purified inclusion bodies was also explored by following the protocol reported by Carlomagno et al (40), whereby a wide array of refolding conditions were generated by combining different buffers (pH range 5-11) and additives (arginine 0.2-1 M, sucrose 0.2-1 M, glycerol 4-40% and PEG 5-20%), but luciferase activity could not be significantly recovered in any of the conditions screened (data not shown). These results, considered alongside our previous experience expressing these constructs, suggest their expression as soluble protein (as opposed to forming inclusion bodies) is nanobody sequence-dependent. Finally, we attempted the expression of NbON10-NanoLuc in the HEK293T cell line. The sequence was codon-optimized for humans, cloned into the pcDNA3.1(+) vector and transfected to be expressed in HEK293T cells. The nucleotide sequence of the cassette used to produce the chimera is shown in **Supplementary Figure 6** (plasmid available at Addgene ID 198691). In this case, the protein was successfully expressed. Transient expression after 4 days yielded 17.6 mg of purified protein per liter of culture medium (**Figure 3B**).

By constructing titration curves using serially diluted full-length NP, we found that the luminescent assay had a limit of detection of 61 pg/mL, which represents a two-fold improvement in analytical sensitivity compared to the colorimetric assay (**Figure 3C**). Once this was established, we investigated whether the assay could detect the Omicron variant NP, as this is the most extensively mutated and currently dominant variant, accounting for nearly 100% of SARS-CoV-2 sequences shared on GISAID as of April 2023 (41). We tested the antigen-capture assay’s ability to detect a mutated NP (Acro Biosystems, Cat. No. NUN-C52Hx) shared by the most dominant subvariants as of April 2023, including BA.5, BQ.1, BQ.1.1 and XBB (containing the mutations P13L, Δ31-33, E136D, R203K, G204R, S413R) (42), and found that the assay was able to successfully detect it and produced similar readouts to the reference NP (**Figure 3D**). This result was expected since NP, being subject to low selective pressure in comparison with the surface receptor-binding Spike protein, has accumulated relatively few mutations.

The luminescent assay’s performance in detecting NP in clinical specimens was initially evaluated using the same set of samples previously used to evaluate the colorimetric assay (n=19 positive samples and n=10 negative samples) (**Figure 3E**). Similarly, a cutoff value was established by constructing a ROC curve and setting the specificity as 100.0% (95% CI: 69.2%-100.0%; **Figure 3F**). With this sample set, the sensitivity was determined to be 84.2% (95% CI: 60.4%-96.6%), with 16/19 positive samples above the cutoff point.

### 3.5 The assay demonstrated high levels of specificity and sensitivity when tested with a large panel of clinical samples

**Figure 4:**
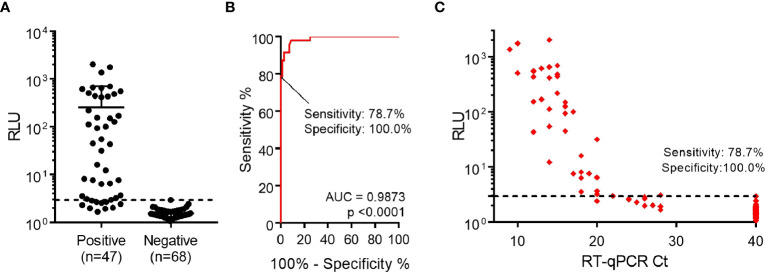
Detection of nucleocapsid protein from nasopharyngeal swab specimens. **(A)** Performance of luminescent antigen-capture ELISA with nasopharyngeal swab samples classified as positive (n=47) or negative (n=68) by prior RT-qPCR analysis. The cutoff value shown (dashed line) was determined through a Receiver Operating Characteristic (ROC) curve. **(B)** ROC curve for the luminescent antigen-capture ELISA, based on the sample set described in the previous point. By setting the assay specificity to 100.0%, sensitivity was determined to be 78.7%. **(C)** Readouts from the same sample analysis plotted against Ct values determined by RT-qPCR. The dashed line represents the cutoff value determined through the ROC curve.

Finally, assay performance was evaluated using a larger set of samples (n=47 RT-qPCR-positive and n=68 RT-qPCR-negative samples). These samples represent the epidemiological landscape of March-April 2022 in Uruguay, a period when Omicron BA.1 was already the dominant variant, representing nearly 100% of new cases (33). After samples were analyzed by luminescent ELISA, a ROC curve was constructed and specificity was set as 100.0% (95% CI: 94.7%-100.0%), determining the sensitivity to be 78.7% (95% CI: 64.3%-89.3%), with 37/47 positive samples correctly identified (**Figures 4A, B**). In addition, we observed a correlation between lower RT-qPCR Ct values and higher signals (**Figure 4C**). Notably, when considering the positive samples of Ct <24, assay sensitivity increased to 97.3% (95% CI: 85.8%-99.9%) (**Supplementary Figure 7**).

## 4 Discussion

The spread of SARS-CoV-2 infection precipitated the biggest public health emergency in recent times. New variants have posed increasingly greater challenges to existing immunity, generated either through natural infection or vaccination. In particular, the recent BQ and XBB subvariants have been shown to compromise the effectiveness of existing vaccines, including those that raise immunity against the Omicron BA.5 subvariant (43). Moreover, all available monoclonal antibody therapeutics fail to neutralize them (44). Although new reported cases are declining, the emergence of further new variants remains a possibility, and testing will continue to play an essential role in preventing the spread of COVID-19.

In this work, we aimed to develop an affordable and highly sensitive laboratory antigen test that allows for high-throughput analysis of untreated samples. To this end, high-affinity nanobodies with non-overlapping epitopes were selected from a phage display library constructed from the peripheral blood mononuclear cells of a llama immunized with SARS-CoV-2 NP. This protein has diverged significantly from the NP of other endemic human coronaviruses, presenting 48.5% identity with MERS-CoV, 36.7% with HCoV-HKU1, 28.8% with HCoV-229E, 48.3% with HCoV-NL63 and 38.4% with HCoV-OC43. However, it contains a highly conserved motif (FYYLGTGP) in the N-terminal domain (34, 35), which could be a source of cross-reactivity and compromise the assay’s specificity. For this reason, a truncated version of NP devoid of this region (NPΔ121) was produced, and nanobodies were screened against this antigen as well as full-length NP, with the aim of ensuring that at least one of the nanobodies included in the assay was reactive against this less-conserved fragment. Although we could alternatively have used NPΔ121 for immunization and selection during panning, we decided against it in order to avoid the exclusion of potential high-affinity clones that bind to the more conserved region, or clones that might empirically be proven to constitute favorable pairs to maximize assay sensitivity. This approach proved successful, because of the three nanobodies selected through this strategy, only H4 binds to NPΔ121, while the remaining two (D5 and ON10) only bind to the full-length protein, suggesting their epitopes lie on the more conserved N-terminal region. The inclusion of H4 as a capture nanobody likely contributed to the high specificity observed in the resulting immunoassay. Nevertheless, this does not rule out the possibility of cross-reactivity of our assay with the NP of other endemic human coronaviruses. Unfortunately this could not be assayed, which represents a limitation of this work.

In order to test the applicability of the selected nanobodies for NP detection in a sandwich ELISA format, a colorimetric ELISA was initially developed, in which the detection nanobody was followed by an anti-HA-HRP secondary antibody. This detection system was subsequently replaced by the introduction of a nanobody-NanoLuc luciferase tracer, which reduced the assay time by eliminating the need for a secondary antibody and, more importantly, resulted in a two-fold increase in analytical sensitivity (**Figure 3**). Although this difference was less pronounced than we had anticipated based on previous reports (30), the analysis of nasopharyngeal swab samples using the luminescent assay showed a highly significant improvement in the clinical performance of the test. Notably, when only the samples with Ct <24 were considered, the luminescent assay attained a sensitivity of 97.3%. This is particularly important, since it has been shown that lower Ct values correlate with cell culture positivity and therefore with the presence of viable virus (45, 46). Correctly identifying these samples as positive is key in order to identify patients undergoing the period of highest infectiousness. As illustrated by the ROC curves, the selection of different cutoff values would be possible in order to allow for different sensitivity/specificity trade-offs. The relative advantages and drawbacks of prioritizing either parameter depend on the epidemiological circumstances as well as the testing protocols in place in a given setting (for instance, whether a follow-up RT-qPCR test is required in the case of a negative result).

The emergence of new variants, particularly subvariants of the Omicron lineage, poses a challenge to the effectiveness of vaccines and monoclonal antibody-based therapeutics. These mainly target the surface receptor-binding Spike protein, which is subject to high selective pressure and therefore presents the highest mutation rate among subvariants. NP is not subject to as much selective pressure, one of the characteristics that make it suitable for antigen detection tests. Nonetheless, the mutations that do emerge may affect the performance of diagnostic tests, and indeed it has been shown that for some tests the detection of recombinant NP that contains variant-associated mutations leads to a decrease in sensitivity (47, 48). To ensure that our assay was effective in detecting the circulating variants, we evaluated the performance of our test using recombinant NP of the BA.5 subvariant, which carries mutations shared by current circulating subvariants such as XBB subvariants and found that it generated similar readouts to the reference NP (**Figure 3D**). More importantly, the clinical samples used to evaluate the assay were collected during the period of March-April 2022, when Omicron was already the dominant variant in Uruguay, thus suggesting that the clinical sensitivity reported corresponds to the detection of the currently circulating variant.

A major goal of this study was to develop a reliable SARS-CoV-2 antigen detection assay that could be reproduced by other laboratories. To the best of our knowledge, this is the first report of a fully-recombinant SARS-CoV-2 NP assay which has demonstrated its potential for the analysis of clinical samples and for which protein sequences have been made freely available. Additionally, this development demonstrates the value of using recombinant chimeric tracers, which constitute highly standardized and reproducible immunoassay reagents and contribute to reducing assay times.

## Data availability statement

The original contributions presented in the study are included in the article/**Supplementary Material**. Nucleotide sequencing data presented in the study are deposited in the GenBank repository, accession numbers OQ982374, OQ982375, OQ982376, OQ982377, OQ982378, OQ982379. Further inquiries can be directed to the corresponding authors.

## Ethics statement

Ethical review and approval was not required for the study on human participants in accordance with the local legislation and institutional requirements. Written informed consent from the participants’ legal guardian/next of kin was not required to participate in this study in accordance with the national legislation and the institutional requirements. The animal study was reviewed and approved by Comisión de Ética en el Uso de Animales (CEUA) Zoológico Parque Lecocq, Intendencia Municipal de Montevideo. Written informed consent was obtained from the owners for the participation of their animals in this study.

## Author contributions

PS-S participated in experimental design, performed most of the experiments, analyzed data, and wrote the manuscript draft. CP-R contributed to optimizing the expression and purification of NP. XS performed sample collection. CE performed llama immunization and blood draw. GL and GG-S participated in experimental design and discussion of results and revised the manuscript. GG-S conceived the study and organized the manuscript writing. All authors contributed to the article and approved the submitted version.
