# Supplementary material for: A highly sensitive nanobody-based immunoassay detecting SARS-CoV-2 nucleocapsid protein using all-recombinant reagents
Source: Front Immunol. 2023 Jul 11;14:1220477. doi: 10.3389/fimmu.2023.1220477 (PMC10367427; doi:10.3389/fimmu.2023.1220477)
Supplement: Supplementary file 1 [file DataSheet_1.docx]

Supplementary Material

A highly sensitive nanobody-based immunoassay detecting SARS-CoV-2 nucleocapsid protein using all-recombinant reagents

Paula Segovia-de los Santos^1^, Carolina Padula-Roca^1^, Ximena Simon^2^, Cesar Echaides^3^, Gabriel Lassabe^1*^, Gualberto Gonzalez-Sapienza^1*^

^1^Cátedra de Inmunología, DEPBIO, Facultad de Química, Instituto de Higiene, Montevideo, Uruguay.

^2^ATGen SRL, Montevideo, Uruguay.

^3^Parque Lecocq, IMM, Montevideo, Uruguay.

*** Correspondence:**Gualberto Gonzalez-Sapienza
ggonzal@fq.edu.uy

Gabriel Lassabe
glassabe@fq.edu.uy

# Supplementary Figures


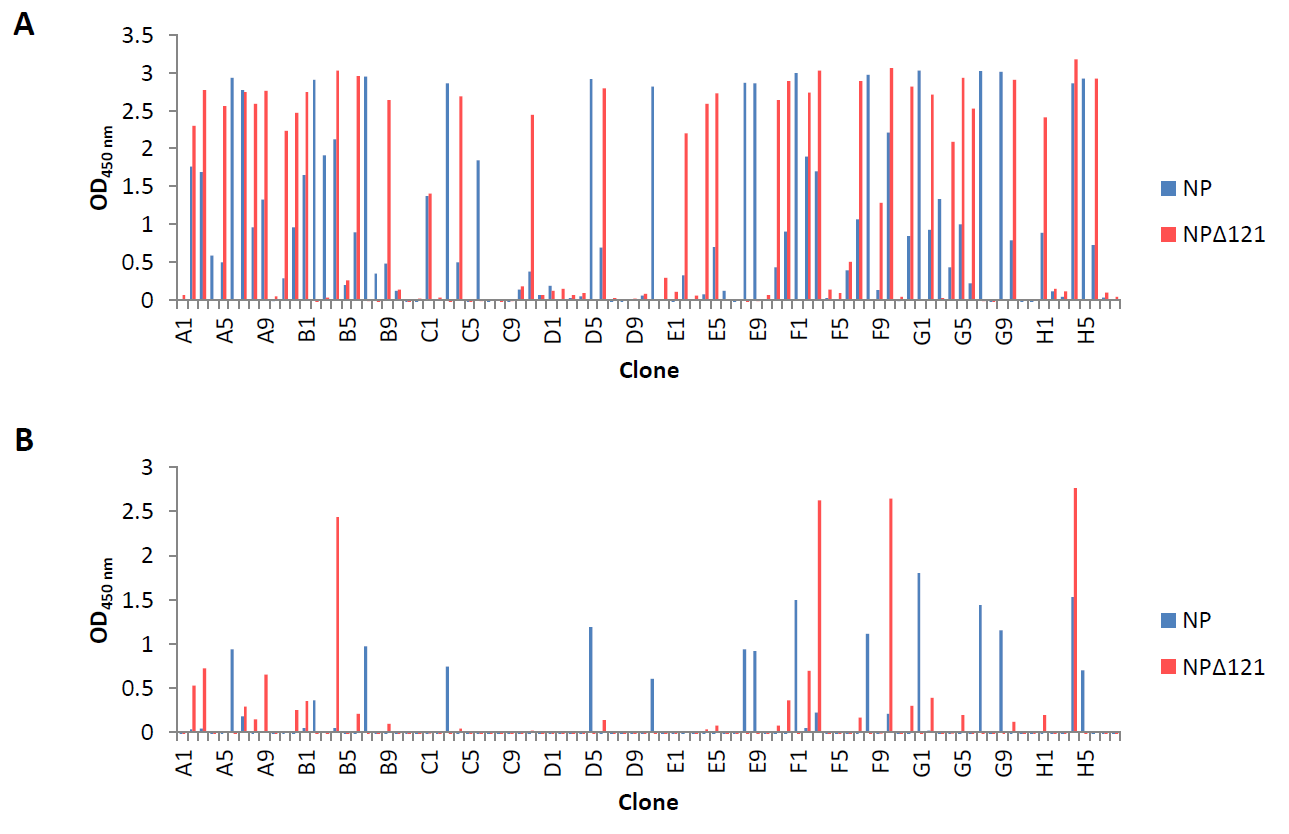


**Supplementary Figure 1. Reactivity of 92 cell lysate supernatants from bacterial cultures of anti-NP clones.** The reactivity was analyzed at a 10^-1^ (A) or 10^-3^ (B) dilution of the supernatants in ELISA plates coated with either full-length NP or streptavidin followed by biotinylated NPΔ121. The bound nanobodies were detected with an HRP-conjugated anti-HA monoclonal antibody.


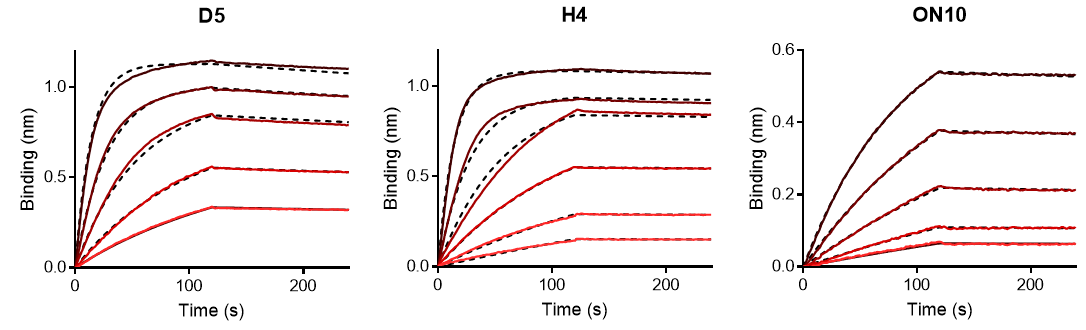


**Supplementary Figure 2. Bio-Layer Interferometry sensograms obtained for the determination of kinetic association and dissociation constants of selected anti-NP nanobodies.** Association and dissociation of each nanobody was measured at concentrations of 400, 200, 100, 50, 25 and 10 nM (the last one was only possible for H4) on amine-reactive biosensors with covalently immobilized full-length NP on the BLItz instrument (ForteBio). Data were globally fitted to a 1:1 binding ratio model (dashed lines).


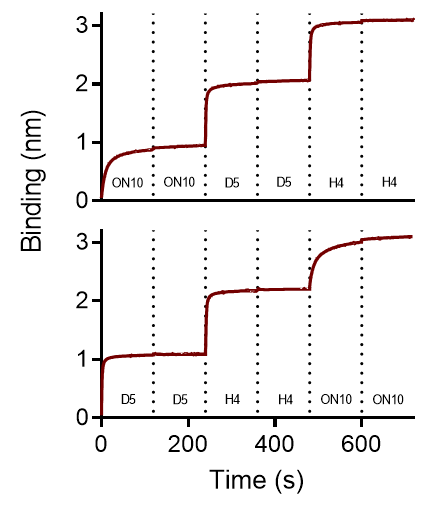


**Supplementary Figure 3. Sensograms for epitope binning of selected nanobodies using the BLItz instrument (ForteBio).** Alternative binding orders for the different nanobodies were analyzed to confirm that epitopes were non-overlapping.

TCTAGAAATAATTTTGTTTAACTTTAAGAAGGAGATATACCATGAAAAAGACAGCTATCGCGATTGCAGTGGCACTGGCTGGTTTCGCTACCGTGGCCCAGGCGGCCATGGCCGAGGTGCAGCTGGTGGAGTCTGGGGGAGGCTTGGTGCAGGCTGGGGGGTCTCTGAGACTCTCCTGTGCAGCCTCTGGATTCCCCTTCAGATTCAATGCCATGGCCTGGTTCCGTCAGGCTCCAGGGAATCAGCGCGAGTTGGTCGCAGGTATTTTCACTACATACGCCACAAATTATGCAGAGTCCGTGAAGGGCCGATTCACCATCTCCAGAGACAACGACAAGAACACGGTCTATCTACAAATGAACAGCCTGAAACCTGAGGACACAGGCGTCTATTACTGTAATGCAGCCTATGTCAGTACAAGTATTTGGCGCCCACTGTATGACGACTGGGGCCAGGGGACCCAGGTCACCGTCTCCTCAGGCCAGGCCGGCCAAGGTGGCGGTCTGAACGACATCTTCGAGGCTCAGAAAATCGAATGGCACGAACTCGAG

**Supplementary Figure 4. Nucleotide sequence of the cassette encoding the capture nanobody H4.** The color code is as follows: yellow, ribosome-binding site (RBS); green, OmpA signal peptide; pink, Nb H4; sky blue, Avi-tag. The sequence was cloned in the pET28a(+) vector between the XbaI and XhoI restriction sites.

TCTAGAAATAATTTTGTTTAACTTTAAGAAGGAGATATACCATGAAAAAGACAGCTATCGCGATTGCAGTGGCACTGGCTGGTTTCGCTACCGTGGCCCAGGCGGCCATGGCCCAGGTGCAGCTGGTGCAGTCTGGGGGAGGCTTGGTGCAGCCTGGGGGTTCCCTGAGACTCTCCTGTGCAGCCTCTGGATTACGTTTGGGTTATTATACCATAGCCTGGTTCCGCCAGGCCCCAGGGAAAGAGCGCGAGGGTATCTCATGTATTAGTAGAAGTGACGGAAGCACATACTATGCAGACTCCGTAAAGGGCCGATTCACCATCTCCACGGACAACGCCGAGAACACGGTATATCTGCAAATGAACAGCCTGAAACCTGAGGACACGGCCGTTTATTACTGTGCAGCGGATTTCGTCCCGGCGTCTCACTGTGCAGTGGCGAATACTAGGGGCTATGACTACTGGGGCCAGGGGACCCAGGTCACCGTCTCCTCAGGCCAGGCCGGCCAGCACCATCACCATCACCATGGCGCATACCCGTACGACGTTCCGGACTACGCTAGCGGATCCTAGTAGCTCGAG

**Supplementary Figure 5.** **Nucleotide sequence of the cassette encoding the detection nanobody ON10 for colorimetric ELISA.** The color code is as follows: yellow, ribosome-binding site (RBS); green, OmpA signal peptide; pink, Nb ON10; orange, 6xHis tag; sky blue, HA-tag. The sequence was cloned in the pET28a(+) vector between the XbaI and XhoI restriction sites.

GGATCCACCATGGAGACAGACACACTCCTGCTATGGGTACTGCTGCTCTGGGTTCCAGGTGCCACTGGTGACGCGGCCCAGGCGGCCATGGCCCAGGTTCAACTGGTACAGAGCGGAGGGGGTCTTGTACAACCTGGAGGATCACTCAGACTGTCTTGCGCTGCGTCTGGGCTGCGACTTGGATATTATACCATAGCCTGGTTCAGACAGGCACCTGGAAAGGAGCGGGAGGGAATTAGTTGCATTAGTAGATCCGACGGGAGCACGTATTACGCCGACTCCGTCAAAGGTCGATTTACAATATCCACGGACAACGCAGAGAACACAGTCTACCTCCAAATGAATAGTCTGAAACCAGAGGACACAGCCGTGTATTACTGCGCCGCAGACTTTGTCCCAGCCAGCCATTGTGCAGTAGCAAACACACGCGGGTATGATTATTGGGGGCAAGGTACCCAGGTCACGGTCAGCTCAAAAGGCCAGGCCGGCCAGGGGGGAGGTGGTTCTGGGGGTGGAGGCTCAGGCGGAGGAGGATCAGAATTCATGGTGTTTACCCTGGAAGATTTCGTCGGTGATTGGCGCCAGACAGCAGGATACAATTTGGATCAAGTGCTTGAACAAGGGGGCGTGAGTAGTCTCTTCCAAAATCTGGGTGTTTCAGTTACGCCTATCCAACGCATTGTACTGAGTGGCGAAAACGGCTTGAAAATTGACATCCACGTCATCATTCCCTACGAAGGACTCAGTGGCGACCAAATGGGGCAAATAGAAAAGATTTTTAAGGTAGTTTACCCCGTGGATGACCACCATTTTAAAGTAATTCTCCATTACGGAACGTTGGTCATAGATGGTGTTACACCCAACATGATAGACTACTTTGGCCGCCCTTACGAGGGCATTGCCGTGTTTGACGGGAAGAAAATTACAGTGACTGGAACATTGTGGAATGGTAATAAAATAATCGACGAACGCCTCATTAACCCAGATGGTTCCCTTCTGTTCCGCGTGACGATAAACGGAGTCACTGGCTGGAGACTGTGTGAGCGCATCTTGGCGACCGGTCTCGAGGACGACGACGACAAGCACCATCACCATCACCATGGGGGCGGAGGCTCTGCTTGGAGTCATCCACAATTCGAAAAAGGTGGAGGTTCCGGAGGTGGATCGGGAGGTTCTGCCTGGTCTCATCCACAATTTGAGAAGGCTAGCGGGGGGTAATAGGCGGCCGCTTTAAATCTAGA

**Supplementary Figure 6. Nucleotide sequence of the cassette encoding the detection nanobody ON10 fused to NanoLuc.** The color code is as follows: green, Igκ leader; pink, Nb ON10; lilac, NanoLuc; orange, 6xHis tag; sky blue, Twin-Strep-tag. The sequence was cloned in the pcDNA3.1(+) vector between the BamHI and XbaI restriction sites.


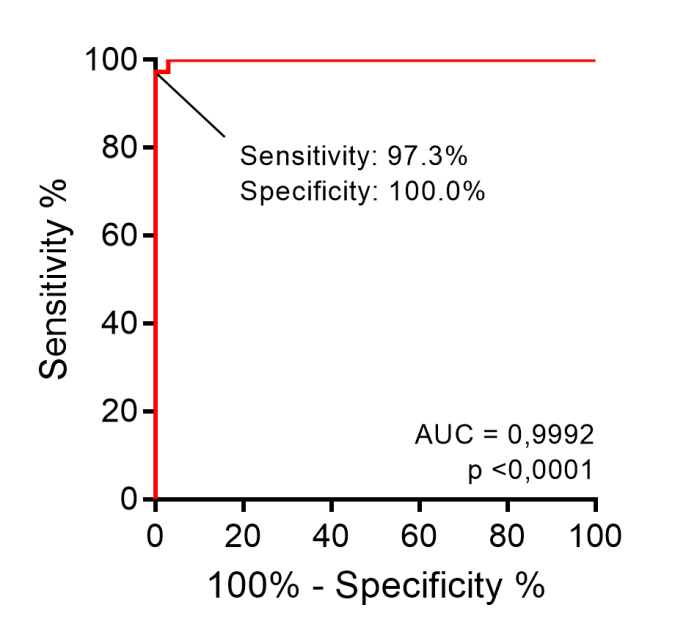


**Supplementary Figure 7. Receiver Operating Characteristic (ROC) curve for the luminescent antigen-capture ELISA for positive samples of Ct <24.** By setting the assay specificity to 100.0%, sensitivity was determined to be 97.3%.
